# Herbal medicine (zhishi xiebai guizhi decoction) for unstable angina: Protocol for a systematic review and meta-analysis

**DOI:** 10.1097/MD.0000000000013965

**Published:** 2018-12-28

**Authors:** Yong Tang, Hairong Cai, Zhenye Zhan, Yajie Luo, Yonglian Huang, Dongjie Chen, Bojun Chen

**Affiliations:** a Department of Internal medicine, The Third Affiliated Hospital of Guangzhou University of Chinese Medicine; b The Third Clinical Medical College; c The Second Clinical Medical College, Guangzhou University of Chinese Medicine; d Department of Emergency, Panyu District Central Hospital of Guangzhou; e Department of Critical Care Medicine, Shenzhen Hospital of Beijing University of Chinese Medicine, Shenzhen; f Department of Emergency, The Second Affiliated Hospital of Guangzhou University of Chinese Medicine, Guangzhou, Guangdong Province, China.

**Keywords:** protocol, systematic review, unstable angina, zhishi xiebai guizhi decoction

## Abstract

**Background:**

Unstable angina (UA) is a clinically common coronary heart disease. Zhishi xiebai guizhi decoction (ZXGD) has been widely used in the management of UA, although its effective evidence is not clear and there is no systematic review regarding its efficacy and safety. Therefore, we conduct this systematic review protocol to evaluate the efficacy and safety of ZXGD in the treatment of UA.

**Methods:**

We will search the following electronic databases: Cochrane Library, Web of Science, PubMed, EBASE, Springer, WHO International Clinical Trial Registration Platform, China Biomedical Literature Database, China National Knowledge Infrastructure, Chinese Scientific Journal Database (VIP), and Wan-fang database from their inception to October 2018. Only randomized controlled trials (RCTs) published in English and Chinese will be included. Continuous data will be expressed as mean difference or standard mean difference, and dichotomous data relative as risk. Study selection, data extraction, and assessment with risk of bias and data analysis will be performed by two independent authors. RevMan software version 5.3 will be used for meta-analysis.

**Results:**

This study will provide high-quality evidence of ZXGD in the treatment of UA from the following aspects, including clinical efficacy, blood lipids, Seattle angina scale, electrocardiogram improvement, ST-segment depression, left ventricular ejection fraction, angina duration, and adverse events.

**Conclusion:**

This systematic review will provide a basis for judging whether ZXGD is an effective intervention for UA or not.

**PROSPERO registration number:**

PROSPERO CRD 42018115528.

## Abstract 2

Supplemental Digital Content is available in the text

## 1 Introduction

Unstable angina (UA) is one serious type of coronary heart disease between stable angina and acute myocardial infarction (AMI), with an abrupt onset, complex condition, and extraordinarily rapid progression, which can easily evolve into AMI and sudden cardiac death.^[1,2]^ UA threatens the life safety of patients seriously with high disability rate and mortality.^[3–5]^ With the development of social economy, the change of residents’ lifestyle, the aging of population structure, the increase of life pressure and the reduction of sports activities, the incidence rate, and mortality rate are increasing year by year, resulting a threat to human life and health. In China, cardiovascular mortality remains the highest, higher than cancer and other diseases.^[6,7]^ The guidelines for the management of UA recommend nitrates, β-blockers, and angiotensin-converting enzyme inhibitors.^[8]^ There is definite effect for modern medicine in treating UA, however, adverse events are still inevitable. Long-term use of nitrate will more likely result in tolerance, headache, and facial flushing,^[9,10]^ and β-blockers may cause a great risk of rebound when they stop suddenly.^[11]^

Traditional Chinese medicine has been widely accepted and applied in practice in China.^[12]^ Zhishi xiebai guizhi decoction (ZXGD) is often used in the management of UA,^[13–15]^ however, but the sample size of randomized controlled trials (RCTs) is small and there is no systematic review regarding its efficacy and safety. Based on the evidence-based medicine method, this systematic review will collect literatures of ZXGD in UA to carry out a meta-analysis of its effectiveness and safety, in order to provide reference for the clinical application.

## 2 Methods

### 2.1 Inclusion criteria for study selection

#### 2.1.1 Types of studies

Only RCTs of ZXGD in the treatment of UA published in Chinese and English, whether blinded or not, will be included. Non-RCTs, animal experiments, case reports, and reviews will be excluded.

#### 2.1.2 Types of patients

Patients with UA (18 years of age and older) must meet the diagnostic criteria established by 2011 ACCF/AHA Focused Update Incorporated Into the ACC/AHA 2007 Guidelines for the Management of Patients With Unstable Angina/Non–ST-Elevation Myocardial Infarction.^[8]^ There is no limitation to patient's age, gender, race, nationality, and comorbidity. Patients with severe liver and kidney dysfunction, AMI, viral myocarditis, mental illness and related drug allergy will be excluded.

#### 2.1.3 Types of interventions

The patients in the experimental group must have been treated with conventional agents (aspirin and other anti-platelet, statin and other lipid-lowering drugs, β-blockers, nitrates, and other basic treatments for coronary heart disease) recommended by guideline^[8]^ combined with ZXGD. The patients in the control group treated with only the same conventional agents or combined with placebo. It is not limited to dose and courses. The follow-up time was ≥6 months. Other complications such as hypertension, diabetes were also treated.

#### 2.1.4 Types of outcome measures

##### 2.1.4.1 Primary outcomes

The primary outcomes are improvement of symptoms of angina pectoris: markedly effective: angina pectoris disappeared primarily, or the number and duration of UA decreased by more than 80%; effective: the number of UA reduced by 50% to 80%; invalid: the number of UA reduced less than 50% or increased, and duration prolonged.

##### 2.1.4.2 Secondary outcomes

The secondary outcomes includes electrocardiogram improvement, ST-segment depression, left ventricular ejection fraction, ventricular stroke volume, angina pectoris, angina duration, Seattle angina scale, blood lipid levels (total cholesterol, triglyceride, low-density lipoprotein, high density lipoprotein), adverse events.

### 2.2 Search methods for the identification of studies

We will search the following electronic databases: Cochrane Library, Web of Science, PubMed, EBASE, Springer, WHO International Clinical Trial Registration Platform, China Biomedical Literature Database, China National Knowledge Infrastructure, Chinese Scientific Journal Database (VIP), and Wan-fang database from their inception to October 2018, using a combined text word and heading search strategy. The search terms include: ZXGD, UA, and RCTs. The strategy for searching the PubMed will be shown as an example in Appendix A (Supplemental Appendix A), and modified by using other databases.

#### 2.2.1 Searching other resources

Meanwhile, we will also search for relevant references in the literature, conference papers, and dissertations to avoid missing grey literature manually. In addition, relevant unpublished research results will be requested from pharmaceutical manufacturers.

### 2.3 Data collection and analysis

#### 2.3.1 Selection of studies

**Figure 1:**
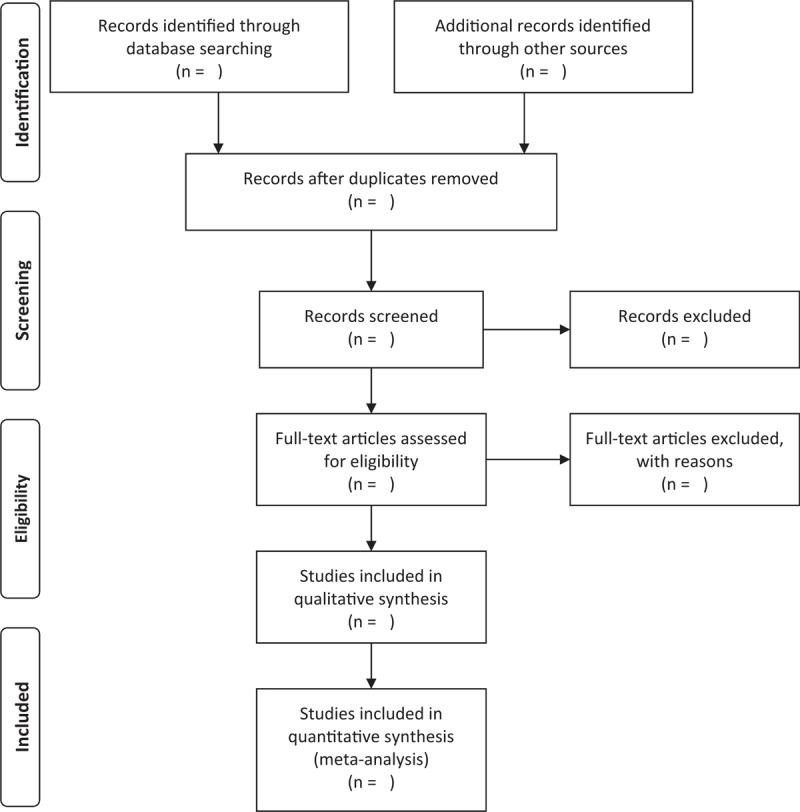
Preferred reporting items for systematic review and meta-analysis (PRISMA) flow chart.

The results will be exported to the Endnote referencing software (version 9.0, Thomas Reuters, CA) and duplicate studies will be excluded. First, the two independent authors will read the titles and abstracts to exclude the trials that do not meet the inclusion criteria. Second, the two independent authors will read the full text of the literature to determine whether to include them or not. Finally, two cross-checking the results will be conducted. If there are any disagreements, they may be resolved through discussion or by the third author. The process of studies selection and meta-analysis is presented in an adapted preferred reporting items for systematic review and meta-analysis flow diagram (Fig. 1).

#### 2.3.2 Data extraction and management

Data extraction and management will be carried out independently by two authors. If there is a disagreement, it will be resolved by discussion or consulting by a third author. The extracted information includes: title, author, length of publication, patient type, diagnostic criteria, randomized method, duration of illness, blinding, gender and number of patients, age of patients, interventions, medication cycle, outcome measures, efficacy criteria, adverse events, data loss, selective reporting.

#### 2.3.3 Assessment of risk of bias in included studies

The risk of bias will be assessed according to the bias risk assessment tool provided by Cochrane Reviewer's Handbook V.5.3, including: random sequence generation, allocation concealment, blinding of the participants and personnel, blinding of the outcome assessments, incomplete outcome data, selective reporting, and other sources of bias. The methodology for each item is graded as a high risk of bias, low risk of bias, and uncertain bias in accordance with the above 7 aspects. “high risk of bias” means that the random method is incorrect, or allocation concealment not used, or blinding not used, or outcome data missing, or key outcomes excluding, or interest of the researcher and the drug manufacturer, so on. “ Uncertain bias” means that the situation is unknown. “Low risk of bias” means that the random method description is detailed and correct, blinding used, ending data complete, and key outcomes reported.

#### 2.3.4 Measures of treatment effect

For dichotomous data, relative risk with 95% confidence intervals will be presented. For continuous data, mean difference or standardized mean difference with 95% confidence intervals will be presented.

#### 2.3.5 Dealing with missing data

If the information is missing, we will contact the author through the letter to obtain it. If more information is not available, data synthesis will be performed based on the available data and we will discuss the impact of the missing data in the text.

#### 2.3.6 Assessment of heterogeneity

The heterogeneity will be assessed by Chi-squared test (a = 0.1) and I^2^ statistic. If *P* > 0.1, I^2^ < 50%, it is considered that there is no statistical heterogeneity or heterogeneity is small. If *P* < 0.1, I^2^ ≥ 50%, it is considered that there is statistical heterogeneity, subgroup analysis or sensitivity analysis will be performed to find the source of heterogeneity.

#### 2.3.7 Assessment of reporting bias

If there are more than 10 articles included, we will use a funnel plot to detect whether there is a publication bias. If the funnel plot is symmetrically funnel-shaped, suggesting that there is no publication bias and conclusion is credible, otherwise, we will treat it with caution.

#### 2.3.8 Data synthesis

Data synthesis will be performed by using RevMan software (version 5.3, The Cochrane Collaboration, Oxford, UK). If the heterogeneity is small or there is no statistical heterogeneity (*P* > 0.1, I^2^ < 50%), the fixed effect model will be used for data analysis. If there is statistical heterogeneity (*P* < 0.1, I^2^≥50%), subgroup analysis or sensitivity analysis will be performed to find whether there is statistical or clinical heterogeneity, the random effects model will be selected in case of eliminating significant clinical heterogeneity. If there is significant clinical heterogeneity, or inability to judge the source of heterogeneity, descriptive analysis will be used.

#### 2.3.9 Subgroup analysis

A subgroup analysis will be used to find the source of heterogeneity according to the evaluation criteria: age, sex, severity of the disease, type, dosage, dosage form, and course.

#### 2.3.10 Sensitivity analysis

If trials data are sufficient, sensitivity analysis to identify whether the conclusions are robust by excluding studies with high risks of bias, studies with missing data, and outliers.

#### 2.3.11 Grading the quality of evidence

The quality of evidence will be evaluated by using the GRADE profiler software (Version 3.6, The GRADE Working Group). The quality of evidence was divided into 4 levels: high, medium, low, and extremely low.

## 3 Discussion

UA is an acute and critical illness in cardiology with characteristics of high hospitalization rate, high disability rate, and high mortality. The pathological mechanism of UA includes rupture of coronary atherosclerotic plaque, incomplete occlusion thrombosis, coronary spasm, which lead to collagen exposure and release more active substances, resulting in platelets adhesion and thrombosis.^[16]^ Clinical researches found that ZXGD should treat UA effectually, and has been widely used in clinical practice. However, there is no systematic review regarding its efficacy and safety so far. Therefore, we will carry out this high-quality systematic review and meta-analysis with the goal of providing a forceful proof of effectiveness and safety for clinical practice, and health policymakers. However, there may be some potential shortcomings in this systematic review. First, it only includes studies published in English and Chinese, which may increase the bias. Second, there may be a heterogeneity risk due to different nationalities, different doses of herbs, and the age of the patient and the small sample.

## Author contributions

**Conceptualization:** Bojun Chen.

**Data curation:** Hairong Cai, Zhenye Zhan.

**Funding acquisition:** Bojun Chen.

**Investigation:** Yonglian Huang.

**Software:** Yajie Luo.

**Supervision:** Bojun Chen.

**Validation:** Dongjie Chen.

**Writing – original draft:** Yong Tang.

## Supplementary Material

Supplemental Digital Content
