# Supplementary material for: Herbal medicine (zhishi xiebai guizhi decoction) for unstable angina: Protocol for a systematic review and meta-analysis
Source: Medicine (Baltimore). 2018 Dec 28;97(52):e13965. doi: 10.1097/MD.0000000000013965 (PMC6314720; doi:10.1097/MD.0000000000013965)
Supplement: Supplemental Digital Content [file medi-97-e13965-s001.docx]

**Appendix A.**

***Search strategy used in PubMed database***

#1 unstable angina OR Anginas, Unstable OR Angina Pectoris, Unstable OR Angina Pectori, Unstable OR Unstable Angina Pectori OR Unstable Angina Pectoris OR Angina at Rest OR Angina, Preinfarction OR Anginas, Preinfarction OR Preinfarction Angina OR Preinfarction Anginas OR Myocardial Preinfarction Syndrome OR Myocardial Preinfarction Syndromes OR Preinfarction Syndrome, Myocardial OR Preinfarction Syndromes, Myocardial OR Syndrome, Myocardial Preinfarction OR Syndromes, Myocardial Preinfarction

#2 Zhishi XieBai Guizhi decoction OR zhi shi xie bai gui zhi decoction OR zhishi xiebai gui tang OR zhishi xiebai guizhi yin

#3 Randomized controlled trial OR clinical study OR Clin-ical Trial OR Controlled study OR Controlled Trial OR Random*Control* study OR random* Control* Trial

#1 AND #2 AND #3
